# Thank you to our reviewers 2023

**DOI:** 10.18849/ve.v9i1.685

**Published:** 2024-03-28

**Authors:** Kit Sturgess+

**Keywords:** THANK YOU, PEER-REVIEW, REVIEWERS

## Abstract

2024 has started with a bang or perhaps more accurately floods, snow and ice. The world remains an uncertain place with the conflict in Ukraine and Gaza as well as tension around the Korean peninsula, Sea of Japan and Taiwan. Economically 2023 was a very hard year with inflation exceeding wage increases making it hard for animal carers, with food banks in the UK seeing an increasing demand for pet food. Thankfully inflation is falling and the economic outlook, although not rosy, is perhaps not as gloomy as originally forecast. The big stories in publishing for 2023 were the explosion of large language models (LLM) like ChatGPT and their impact on publishing (we now have a policy on the use of artificial intelligence (AI) in papers submitted to *Veterinary Evidence*) and the burgeoning challenge of paper mills (organisations that produce and sell authorship of fabricated or manipulated manuscripts which resemble genuine legitimate research) that has led to some journals losing their citation.

*Veterinary Evidence *continues to grow and flourish with increased visits to our website and a regular stream of articles being published. Good governance and ethical publication remain a key focus of the Board that has been strengthened by a number of new members, bringing a broader range of expertise and experience. Our Student Awards continue to be a success and I would like to thank the whole team of staff and reviewers for turning round these submissions so quickly. I had the pleasure of talking to the prize-winning authors and it was great to hear their enthusiasm, but also how much they have valued the help and support from the journal.

Our new submission system, Editorial Manager, has been installed and is working well providing better insight into how the publishing process is working for us. It has been a learning experience for all and like any new system there are a few areas where we can further improve the author and reviewer experience. Thanks to the hard work of staff, reviewers and Associate Editors, there are now no papers left in the old OJS system. The journal has strived to make the reviewing process as straightforward as possible with a prereview checklist to ensure papers are ready for review. A mentoring system is up and running for new reviewers open to all who may be interested in contributing to the publication of veterinary literature. *Veterinary Evidence* has been working hard on our next review date policy and processes so some of our early Knowledge Summaries can be updated as new literature becomes available. We are also preparing to make an application for citation in PubMed Central to increase the visibility of *Veterinary Evidence’s* content.

None of this would be possible without the input from our volunteers and I would like to take this opportunity to say a huge thank you to all our reviewers, Associate Editors and everyone who has served on the Board this year for their tireless efforts on behalf of *Veterinary Evidence* without your support the journal could not exist. This year the Board has again had three online meetings, which have been essential in helping develop the strategy for *Veterinary Evidence*; such as our response to LLM, graphic content and further work on which types of evidence can be included in our Knowledge Summaries. On a personal note, my time as Editor-in-Chief is drawing to a close. It has been a great 3 years plus and I have had incredible support from everyone at *Veterinary Evidence* which has made my role so much easier and more enjoyable.

Once again, a massive thank you to everyone who works for and supports *Veterinary Evidence*, wishing you all a safe and productive 2024.

Kit Sturgess

Editor-in-Chief

2024 has started with a bang or perhaps more accurately floods, snow and ice. The world remains an uncertain place with the conflict in Ukraine and Gaza as well as tension around the Korean peninsula, Sea of Japan and Taiwan. Economically 2023 was a very hard year with inflation exceeding wage increases making it hard for animal carers, with food banks in the UK seeing an increasing demand for pet food. Thankfully inflation is falling and the economic outlook, although not rosy, is perhaps not as gloomy as originally forecast. The big stories in publishing for 2023 were the explosion of large language models (LLM) like ChatGPT and their impact on publishing (we now have a policy on the use of artificial intelligence (AI) in papers submitted to *Veterinary Evidence*) and the burgeoning challenge of paper mills (organisations that produce and sell authorship of fabricated or manipulated manuscripts which resemble genuine legitimate research) that has led to some journals losing their citation.

*Veterinary Evidence *continues to grow and flourish with increased visits to our website and a regular stream of articles being published. Good governance and ethical publication remain a key focus of the Board that has been strengthened by a number of new members, bringing a broader range of expertise and experience. Our Student Awards continue to be a success and I would like to thank the whole team of staff and reviewers for turning round these submissions so quickly. I had the pleasure of talking to the prize-winning authors and it was great to hear their enthusiasm, but also how much they have valued the help and support from the journal.

Our new submission system, Editorial Manager, has been installed and is working well providing better insight into how the publishing process is working for us. It has been a learning experience for all and like any new system there are a few areas where we can further improve the author and reviewer experience. Thanks to the hard work of staff, reviewers and Associate Editors, there are now no papers left in the old OJS system. The journal has strived to make the reviewing process as straightforward as possible with a prereview checklist to ensure papers are ready for review. A mentoring system is up and running for new reviewers open to all who may be interested in contributing to the publication of veterinary literature. *Veterinary Evidence* has been working hard on our next review date policy and processes so some of our early Knowledge Summaries can be updated as new literature becomes available. We are also preparing to make an application for citation in PubMed Central to increase the visibility of *Veterinary Evidence’s* content.

None of this would be possible without the input from our volunteers and I would like to take this opportunity to say a huge thank you to all our reviewers, Associate Editors and everyone who has served on the Board this year for their tireless efforts on behalf of *Veterinary Evidence* without your support the journal could not exist. This year the Board has again had three online meetings, which have been essential in helping develop the strategy for *Veterinary Evidence*; such as our response to LLM, graphic content and further work on which types of evidence can be included in our Knowledge Summaries. On a personal note, my time as Editor-in-Chief is drawing to a close. It has been a great 3 years plus and I have had incredible support from everyone at *Veterinary Evidence* which has made my role so much easier and more enjoyable.

Once again, a massive thank you to everyone who works for and supports *Veterinary Evidence*, wishing you all a safe and productive 2024.

Kit Sturgess

Editor-in-Chief

Fergus Allerton

Anthi Anatolitou

Duncan Barnes

Dan Batchelor

Nicola Bates

Livia Benato

Cameron Black

Caroline Blundell

Carlotta Bocci

Marlies Bohm

Louise Buckley

John Campbell

Tom Candy

Martha Cannon

James Carmalt

John Carr

Marios Charalambous

John Chitty

Louise Clark

Ana Cloquell Miro

Francesca Compostella

Alessia Cordella

Claire Corridan

Gwen Covey-Crump

James Crabtree

MV Cruyningen

Amanda Curtis

Ajoke Ehimiyein

Salih Eminaga

Debbie Emmerson

Terry Emmerson

Sally Everitt

Marco Fantinati

Erik Fausak

John Fishwick

Myra Forster-van-Hijfte

Elisabeth Fox

Mary Fraser

Ben Garland

Barbara Glanemann

Eloi Guarnieri

Sandra Guillen

Simon Hagley

Emily Hall

Jennifer Hamlin

Katherine Hart

Karen Humm

Jo Ireland

Clare Margaret Knottenbelt

David Leicester

Theofanis Liatis

Loni Loftus

Richard Malik

Miltiadis Markou

Nina Meyerhoff

Antonio Mollo

Andy Morris

Joanna Morris

John Munday

Sue Murphy

Jane Oatley

Conor O'Halloran

Matthew Oxford

Carolina Palacios Jimenez

Valentin Parmen

Christopher Parratt

Peter Plate

Brian Pound

Marc Raffe

David Ramey

Ian Ramsey

Adam Revitt

Emma Robertson

Janet Rodgers

Lynda Rutherford

Ian Self

Francisco Silveira

Bruce Smith

Eva Spada

Adam Swallow

Kelly Tait

Emma Tallini

Anna Tauro

Theodora Tsouloufi

Alexandru Tutunaru

Enzo Vettorato

Kate White

Nayana Wijayawardhane

Sophie Wyatt

## ORCiD

Kit Sturgess: https://orcid.org/0000-0002-3402-5113

## Contribute to the evidence

There are two main ways you can contribute to the evidence base while also enhancing your CPD:

- Tell us your information need
- Write a Knowledge Summary

Either way, you will be helping to add to the evidence base, and strengthen the decisions that veterinary professionals around the world make to give animals the best possible care. Learn more here: https://veterinaryevidence.org/index.php/ve/guidelines-for-authors

## Disclaimer

Knowledge Summaries are a peer-reviewed article type which aims to answer a clinical question based on the best available current evidence. It does not override the responsibility of the practitioner. Informed decisions should be made by considering such factors as individual clinical expertise and judgement along with patient’s circumstances and owners’ values. Knowledge Summaries are a resource to help inform and any opinions expressed within the Knowledge Summaries are the author's own and do not necessarily reflect the view of the RCVS Knowledge. Authors are responsible for the accuracy of the content. While the Editor and Publisher believe that all content herein are in accord with current recommendations and practice at the time of publication, they accept no legal responsibility for any errors or omissions, and make no warranty, express or implied, with respect to material contained within. For further information please refer to our Terms of Use.

